# Efficacy of modified thoracoabdominal nerves block through perichondrial approach in open gynecological surgery: a prospective observational pilot study and a cadaveric evaluation

**DOI:** 10.1186/s12871-022-01652-2

**Published:** 2022-04-15

**Authors:** Nobuhiro Tanaka, Takanori Suzuka, Yuma Kadoya, Naoko Okamoto, Mariko Sato, Hideaki Kawanishi, Cho Azuma, Mayumi Nishi, Masahiko Kawaguchi

**Affiliations:** 1 Department of Anesthesiology, Nara Medical University, 840 Shijo-cho, Kashihara, Nara, 634-8522 Japan; 2 Takatsuki General Hospital, 1-3-13, Kosobe-cho, Takatsuki, Osaka, 569-1192 Japan; 3 Department of Anatomy and Cell Biology, Nara Medical University, 840 Shijo-cho, Kashihara, Nara, 634-8521 Japan

**Keywords:** Modified thoracoabdominal nerves block through perichondrial approach; M-TAPA, Gynecological surgery, Analgesia, Peripheral nerve block

## Abstract

**Background:**

Modified thoracoabdominal nerves block through perichondrial approach (M-TAPA) was first described as a peripheral nerve block by Tulgar in 2019. This technique provides an analgesic effective range from Th7–11 with a single puncture per side. Although the efficacy and effective duration of M-TAPA have been reported, further examination is required. Therefore, this study aimed to evaluate the analgesic range and effective duration of M-TAPA in open gynecologic surgery.

**Methods:**

Following approval, 10 adult female patients scheduled for open radical hysterectomy via a vertical incision or laparotomy using a midline incision from under the xiphoid process to the symphysis pubis were enrolled. The primary outcome was the number of anesthetized dermatomes at 2 and 24 h postoperatively. Secondary outcomes included numerical rating scale scores and the total amount of fentanyl used. Cadaveric evaluation was performed to assess the spread of the dye.

**Results:**

The median numbers (interquartile range) of anesthetized dermatomes at 2 and 24 h postoperatively were 6 (5–7) and 6.5 (5–7) in the anterior cutaneous branch area and 5 (4–7) and 7 (5–7) in the lateral cutaneous branch area, respectively. There was an 85% chance of simultaneously acquiring analgesia in areas innervated by Th8–11, including complete block in areas innervated by the anterior cutaneous branches of Th9–10. Cadaveric evaluation showed the spread of the dye in Th8–11.

**Conclusions:**

M-TAPA may have analgesic effects in the areas supplied by the anterior cutaneous branches of Th8–11.

**Trail registration:**

IRB approval (No.2700; registered on July 10, 2020) and registration (UMIN Clinical Trials Registry: UMIN000041137; registered on July 17, 2020).

## Background

The pain after hysterectomy is very uncomfortable for patients, with an average score of 6 out of 10 on the numerical rating scale (NRS) [1]. Furthermore, analgesia in cases of large incisions, such as in radical hysterectomy, including para-aortic lymphadenectomy or omentectomy, is typically challenging. Options for regional anesthesia as an important part of a multimodal analgesic strategy in radical hysterectomy include epidural anesthesia, transverse abdominis plane block (TAPB), rectus sheath block (RSB), and continuous wound infiltration (CWI) analgesia. However, each of these options has its disadvantages. Epidural analgesia has been suggested to be associated with increased 30-day morbidity and length of stay after abdominal hysterectomy for gynecologic malignancy [2]. There are many reports that TAPB is effective in gynecological laparotomy [3, 4]; however, there are concerns that the local anesthetic dose per site is inevitably reduced by multiple punctures in ‘dual’ TAPB, which combines subcostal and lateral TAPB, oblique subcostal TAPB, or RSB. The effectiveness of CWI through multiple-orifice catheters has also been reported [5, 6], but the expected even distribution of local anesthetic solution through each pore may not be guaranteed [7].

.Thoracoabdominal nerves block through perichondrial approach (TAPA) was described in 2019 by Tulgar as a peripheral nerve block that provides effective analgesia between Th5 and Th12 [8]. The original TAPA technique requires double injection in which a local anesthetic is applied to the lower and upper aspects of the chondrium. Tulgar et al. also described a modified TAPA (M-TAPA) technique as a novel technique in which a local anesthetic agent is applied only to the lower surface of the chondrium [9]. M-TAPA may show an extensive analgesic range in front of the abdomen, with a single puncture per side. The efficacy and effective duration of M-TAPA have been reported in several case reports [10, 11] and one research report in laparoscopic surgery [12]; however, this subject requires further investigation, especially in laparotomy.

Therefore, this pilot study aimed to evaluate the analgesic range and effective duration of M-TAPA in open gynecologic surgery.

## Methods

### Study design and patient enrollment

This prospective observational study was approved by the Nara Medical University Ethics Committee, Kashihara, Nara, Japan (No.2700; registered on July 10, 2020) and registered in the UMIN Clinical Trials Registry (UMIN000041137; registered on July 17, 2020) before enrollment commencement. Written informed consent was obtained from all study participants. All methods were carried out in accordance with the Declaration of Helsinki. A total of 10 American Society of Anesthesia class I –III adult (age 18–75 years) patients scheduled for open radical hysterectomy using a vertical incision or laparotomy using a midline incision from under the xiphoid process to the symphysis pubis (ex. the addition of omentectomy) were enrolled. Exclusion criteria included communication difficulty, allergy to local anesthetic agent, skin disease at puncture site, body mass index >35 kg/m^2^, body weight < 42 kg, coagulation disorder (prothrombin time-international normalized ratio > 1.25, activated partial thromboplastin time > 35 s, or platelet <100,000/μL), and insufficient period of antithrombotic drug withdrawal and chronic opioid use (daily use within 1 month preoperatively).

Patients received general anesthesia under standardized monitoring, according to our institutional rules. Anesthesia was maintained by propofol (using target-controlled infusion, according to an electroencephalogram monitor), sevoflurane (1.0–1.5%), or desflurane (4.0–5.0%), based on the discretion of each anesthesiologist and according to the patient’s condition and complications. Remifentanil, fentanyl, and rocuronium were administered by the anesthesiologist in charge, as needed.

**Fig. 1:**
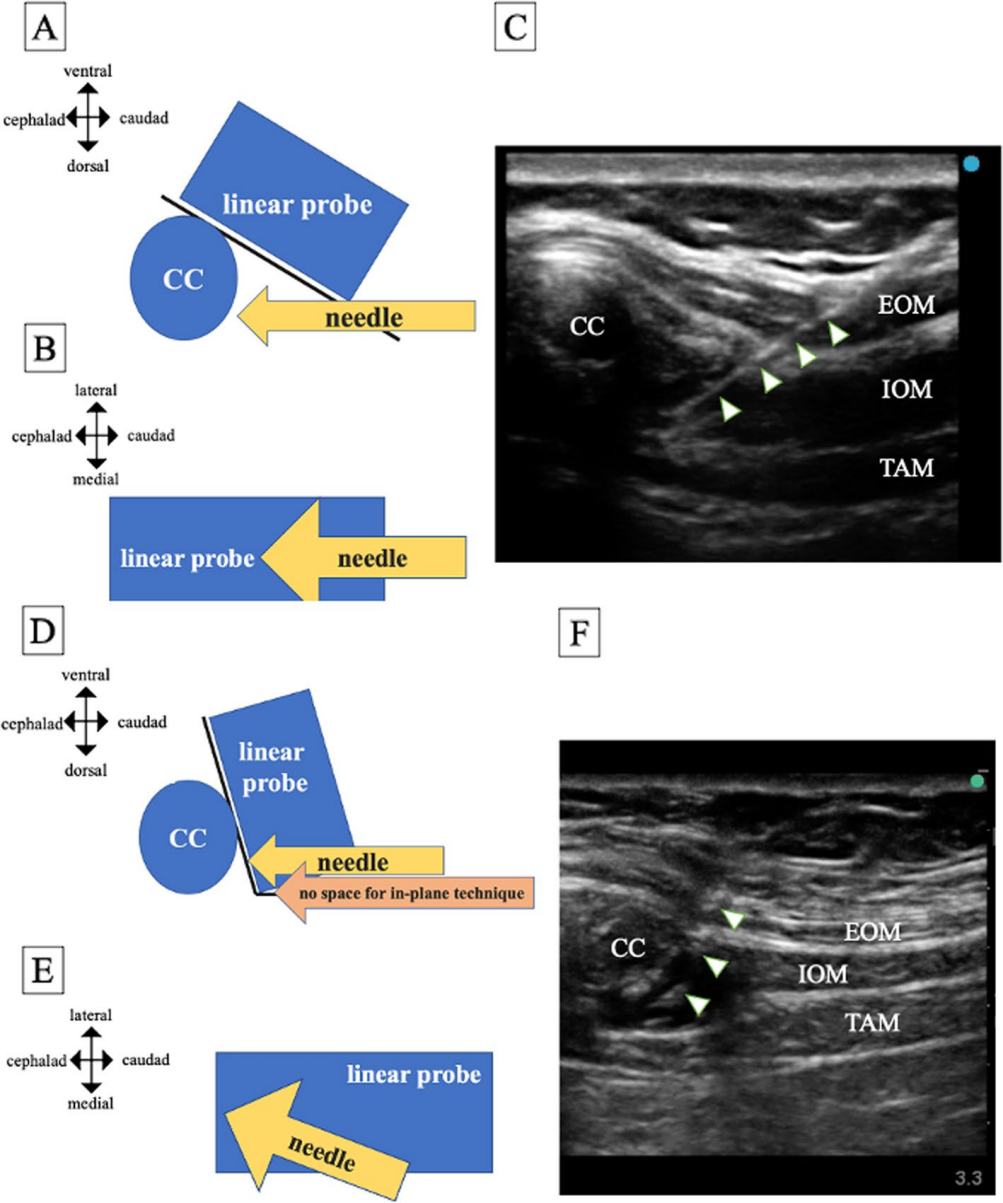
**A**, **B**, and **C** represent the in-plane technique of M-TAPA. **A** Relationship between the costal cartilage (CC), a linear probe for ultrasound, and a needle. **B** Image as seen from above. **C** Ultrasound image of M-TAPA ‘in-plane’ technique. **D**, **E**, and **F** Represent the ‘hybrid’ technique in which the start of puncture is performed like the out-plane technique, but the needle movement is similar to the in-plane technique. **D** and **E** Image seen from the side and above, respectively. **F** Ultrasound image of the M-TAPA ‘hybrid’ technique. CC: costal cartilage, EOM: external oblique muscle, IOM: internal oblique muscle, TAM: transverse abdominis muscle, ‘arrow head’ represent the needle.

Ultrasound-guided M-TAPA was performed using a linear probe (6–13 MHz) of EDGE II (Fujifilm Sonosite, Tokyo, Japan) after anesthesia induction for patients in the supine position. We identified the 10th costal cartilage by the notch between the 9th and 10th costal cartilages, placed a linear probe in the sagittal plane, and angled it deeply to view the lower aspect of the chondrium. A 20-gauge Tuohy needle (UNIEVER disposable nerve blockade needle Huber, UNISIS, Tokyo, Japan) was inserted as much as possible via an in-plane technique. However, in patients with protruding thorax, due to emaciation or pectus carinatum, the caudal end of the probe is deeply dug into the patient’s abdomen, leaving no space for needle insertion for the in-plane technique. In these cases, we performed M-TAPA using a ‘hybrid’ technique in which the puncture is started like the out-plane technique, but the needle movement is similar to the in-plane technique (Fig. 1). After confirming negative aspiration, we applied 0.25% ropivacaine 30 mL (or 25 mL if the patient weighed <50 kg) per side, between the 10th costal cartilage and the transverse abdominis muscle (TAM) at the lower aspect of the chondrium.

All patients were transferred to the ward after their recovery from general anesthesia. An anesthesiologist or a perioperative nurse who belonged to the postoperative care service team assessed anesthetized dermatomes by pinprick tests at 2 and 24 h postoperatively, without the assistance of the anesthesiologist who performed the M-TAPA block. Pinprick tests using 4.0 g Semmes–Weinstein monofilament (SOT-DM06A, SAKAI Medical Co., Ltd., Tokyo, Japan) were performed on the area from Th6 to Th12, based on Keegan and Garrett’s sensory dermatome map, which includes the area 3 cm lateral from the midline incision to avoid wound dressings, to evaluate the anterior cutaneous branch innervation area, and the mid-axillary line to evaluate the lateral cutaneous branch innervation area. The pinprick test was performed in a unified way, from a height of 2.5 cm, lower the monofilament vertically to the examination site for 1.5 s until the filament bends for 1.5 s, and then return the monofilament to its original position for 1.5 s, as per the manufacturing details (0 = loss of pinprick sensation, 1 = decreased pinprick sensation, 2 = normal pinprick sensation). An effective sensory block was defined as a score of 0 or 1.

Intravenous patient-controlled analgesia (IV-PCA) using a mechanical infusion pump (CADD®^□^-Solis 2110, Smiths Medical Japan Ltd., Tokyo, Japan) was started at the time of wound closure, with a setting of 25 μg/h (25 μg bolus and a lock-out time of 10 min) or 20 μg/h (20 μg bolus and a lock-out time of 10 min). Dosing rate was determined by the anesthesiologist in charge to be approximately 0.5 μg/kg/h per body weight. The decision to discontinue IV-PCA was left to the gynecologist. Moreover, the regimen of postoperative analgesia and antithrombotic therapy was ordered by the gynecologists. Acetaminophen (15 mg/kg) was administered every 6 h, starting immediately after the patient’s return to the ward until the postoperative day (POD) 2. In addition, drip infusion of flurbiprofen was administered when the postoperative pain was uncontrollable. Regular oral administration of celecoxib was initiated in the evening of POD 2.

The primary outcome of this study was the number of anesthetized dermatomes at 2 and 24 h postoperatively. The secondary outcomes were NRS scores (0–10, 0: no pain, 10: worst possible pain), intra- and postoperative amount of fentanyl use, occurrence of postoperative nausea and vomiting (PONV) within 48 h postoperatively, the number of additional analgesics used, and the patient satisfaction score (0–10; 0, very unsatisfied; 10, very satisfied).

This study was planned as a pilot study to evaluate the efficacy of M-TAPA in radical hysterectomy. Before the COVID-19 pandemic, we usually recorded 30 cases of radical hysterectomy in our institution yearly; however, for this study, we chose a sample size of 10 patients in consideration of a possible reduction in the number of operations due to the pandemic and the novelty of M-TAPA.

**Fig. 2:**
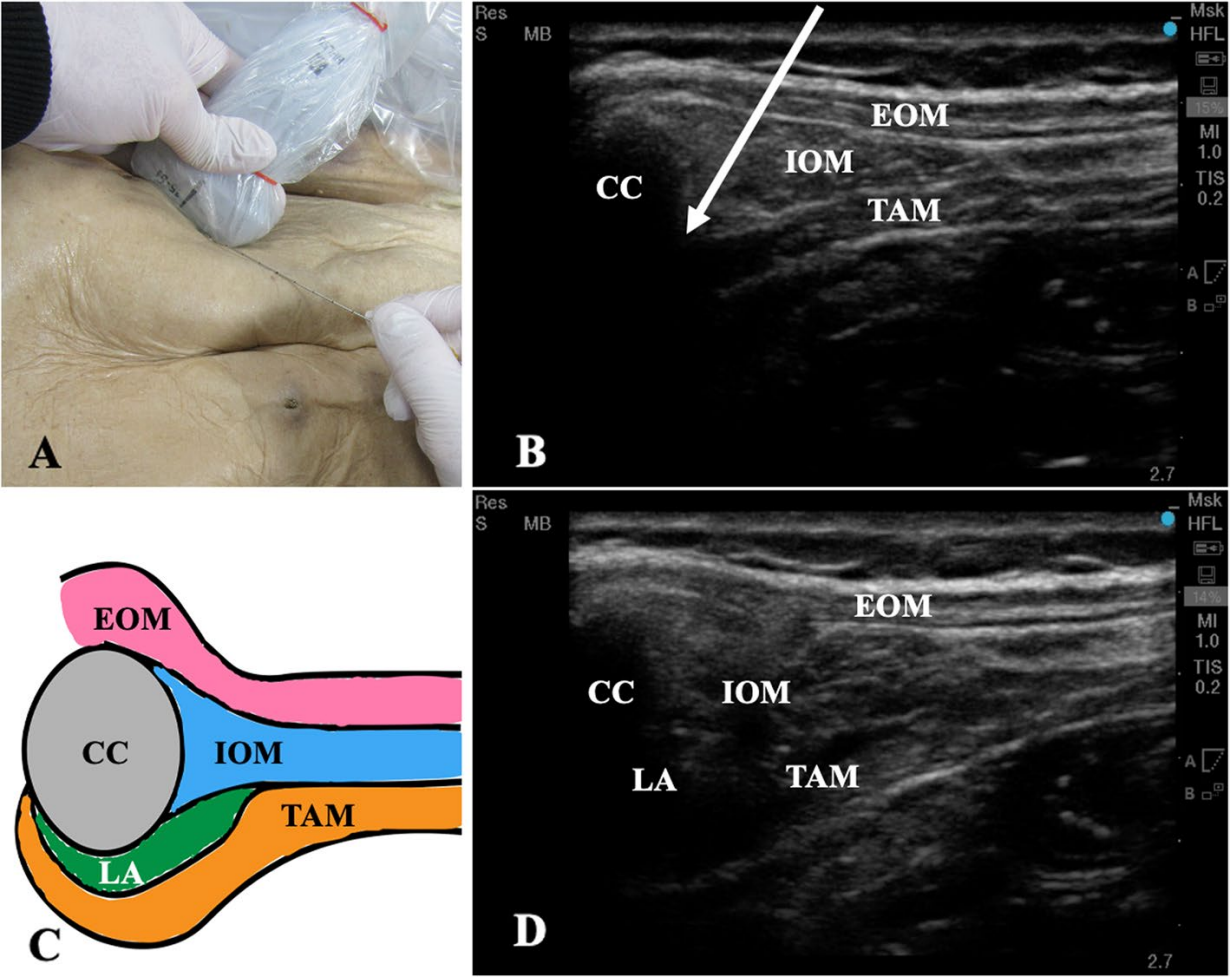
**A** Photograph showing the cadaver, ultrasound transducer, and the needle position for a left-side M-TAPA. **B** Ultrasound image before M-TAPA. **C** Image of successful local anesthetic spread in M-TAPA. We hypothesized that the tunnel structure created by hydrodissection between the costal cartilage and the origin of transverse abdominis muscle and this ‘tunnel’ formed by the two structures leads to the spread of local anesthetic agent higher than Th8, unaffected by the linea semilunaris. **D** Ultrasound image after M-TAPA. CC: costal cartilage, EOM: external oblique muscle, IOM: internal oblique muscle, LA: local anesthetic, TAM: transverse abdominis muscle

Cadaveric evaluation was also performed with the cooperation of members of the Department of Anatomy and Cell Biology, Nara Medical University (Kashihara, Japan). Ultrasound-guided M-TAPA was simulated in four sides in two adult male cadavers with the same procedure as clinical study. All cadavers were embalmed using formalin. After the needle tip was confirmed to be in the appropriate space using ultrasound, 25 mL of a water-soluble dye (Sakura Mat Water Colors Multi; Sakura Color Products Corporation, Osaka, Japan) was injected into cadaver 1, and 30 mL of the dye was injected into cadaver 2 (Fig. 2). The injections were performed by a single anesthesiologist (N.T.), and approximately 30 min after the injections, an anatomist (C.A.) assessed the spread of the dye.

## Results

Fourteen patients were enrolled in this study. Four patients were excluded because of reasons, such as the inability to perform the pinprick test (two patients), due to urostomy associated with combined resection of the bladder (one patient), and colostomy in addition to rectal resection (one patient). Two patients were additionally excluded due to change of operation: one underwent total hysterectomy and bilateral ovarian resection, and the other underwent Pfannenstiel incision.

Patients’ characteristics are presented in Table 1. One patient did not undergo total hysterectomy to maintain fertility, and three patients received an incision above the umbilicus, but not on the xiphoid process because para-aortic lymphadenectomy or omentectomy were not performed.

**Table 1:** Patient characteristics

	All patients (*n*=10)
Age (years), median (IQR)	50.4 (43.5–58.5)
ASA grade (1/2)	3/7
Height (cm), mean (SD)	156±5
Weight (kg), mean (SD)	60±10
Duration of surgery (min), median (IQR)	241 (159–260)
Duration of anesthesia (min), median (IQR)	298 (193–322)
Intraoperative fentanyl use (μg/kg), median (IQR)	7.1 (5.7–9.5)
Time-weighted average of remifentanil use (μg/kg/min), median (IQR)	0.18 (0.15–0.22)
Blood loss (mL), median (IQR)	728 (448–770)
Urine output (mL), median (IQR)	205 (155–270)
Total input (mL), median (IQR)	3000 (2300–3383)
Pre-operative albumin level (mg/dL), mean (SD)	4.3±0.2
Type of surgery	
ATH+BSO+PLA+PALA+OMX	4
ATH+BSO+PLA	3
ATH+BSO+OMX	2
BSO+OMX	1

*ATH* Abdominal total hysterectomy, *BSO* Bilateral salpingo-oophorectomy, *IQR* Interquartile range, *PLA* Pelvic lymphadenectomy, *PALA* Para-aortic lymphadenectomy, *OMX* Omentectomy

**Fig. 3:**
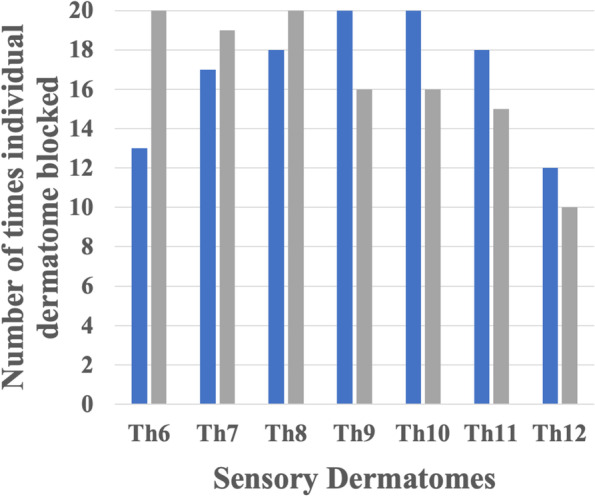
Bar graph showing the number of times each individual sensory dermatome was blocked (Y-axis). X-axis represents sensory dermatomes, from Th6–12, where the intercostal nerve anterior cutaneous branch innervates. Blue bar: 2 h postoperatively, gray bar: 24 h postoperatively.

**Fig. 4:**
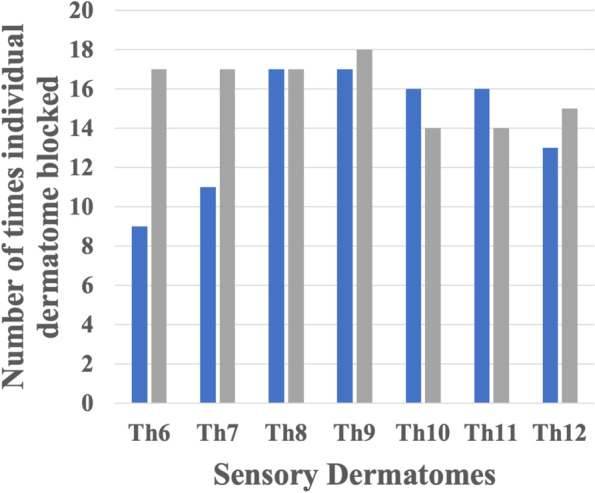
Bar graph showing the number of times each individual sensory dermatome was blocked (Y-axis). X-axis represents sensory dermatomes from Th6–12, where the intercostal nerve lateral cutaneous branch innervates. Blue bar: 2 h postoperatively, gray bar: 24 h postoperatively

The number of times each individual sensory dermatome was blocked in the anterior cutaneous branch and the lateral cutaneous branch are presented in Figs. 3 and 4, respectively.

The median number (interquartile range [IQR]) of anesthetized dermatomes at 2 h postoperatively was 6 (range: 5–7), including complete block in the area supplied by the anterior cutaneous branches of Th9 and Th10. At 24 h postoperatively, the median number (IQR) of anesthetized dermatomes in the area innervated by anterior cutaneous branches was 6.5 (range: 5–7), including a higher blocking rate for Th6–8 (Th6: 100%, Th7: 95%, Th8: 100%). In contrast, the blocking rate for Th12 at 2 and 24 h postoperatively were 50 and 60%, respectively.

The median number (IQR) of anesthetized dermatomes at 2 h postoperatively was 5 (range: 4–7) and at 24 h postoperatively was 7 (range: 5–7) in the lateral cutaneous branch area. Compared with results in the anterior cutaneous branch area, there was no consistent pattern of dermatome anesthesia; effects were observed from Th6 to Th12 in some cases and in some cases, no effect was noted. Other results are presented in Table 2.

**Table 2:** Secondary outcomes

	All patients (*n*=10)
NRS at rest	
2 hours	5 (2–6.5)
24 hours	0.5 (0–2)
48 hours	1 (0–2)
NRS on movement	
2 hours	NA
24 hours	3 (3–5.5)
48 hours	3.5 (3–5)
Total amount of postoperative fentanyl use in μg/kg	
0–12 hours	6.1 (5.1–6.7)
12–24 hours	5.7 (4.6–8.4)
24–36 hours	4.6 (1.9–6.2)
36–48 hours	3.0 (0.6–3.9)
Nausea and vomiting within 48 hours postoperatively (+/-)	4/6
Number of additional analgesic use, n (%)	
Nil	8 (80%)
3 times	2 (20%)
Patient satisfaction	8.5 (8–10)

Data are presented as median (IQR) or mean (SD)

*NRS* Numerical rating scale

NRS scores at rest were 5 (range: 2–6.5), 0.5 (range: 0–2), and 1 (range: 0–2) at 2, 24, and 48 h postoperatively, respectively. NRS scores on movement were 3 (3–5.5) and 3.5 (3–5) at 24 and 48 h postoperatively, respectively. NRS scores on movement at 2 h postoperatively were not available; the patients hardly moved within 2 h postoperatively. PONV occurred in four patients, two of whom were actually vomiting. Regarding patient satisfaction, one patient indicated a score of five points, but all others indicated scores of eight points or more. There was no case of suspected systemic toxicity due to the local anesthetic agent.

**Fig. 5:**
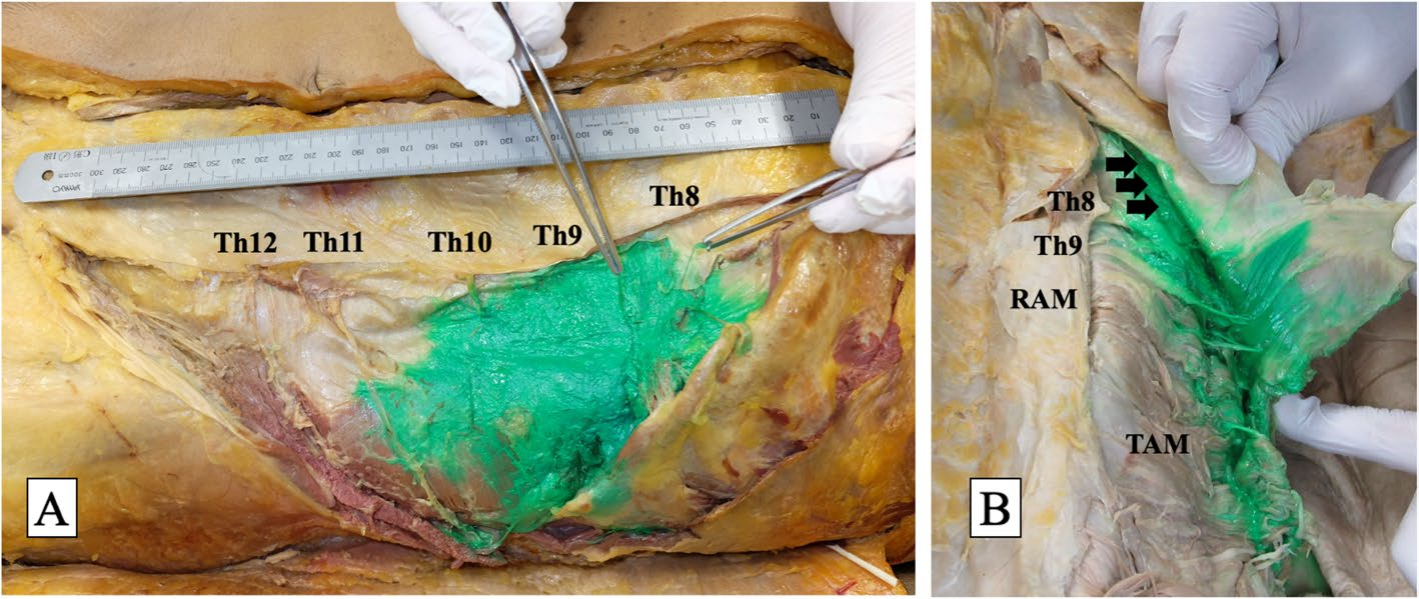
**A** Represents the spread of the dye in the cadaver (the left side of cadaver 2). **B** Represents the spread of the dye between the costal cartilage and the transverse abdominis muscle. Black arrow represents the dye in the ‘tunnel structure’

Spread of the dye was confirmed in Th8–11 in both cadavers; however, we could not confirm the spread of the dye in Th6, Th7, and Th12. We administered 25 mL of the dye in cadaver 1 and confirmed the results as mentioned above. We decided to administer 30 mL of the dye in cadaver 2 to examine whether dye spread could be expanded by an increase in the volume of the local anesthetic agent. Cadaver 2 had a surgical scar at the right flank, which was assumed to have occurred after renal surgery. Caudal spread of the dye was noted to stop along the surgical scar. The spread of the dye on the left side of cadaver 2 is shown in Fig. 5.

## Discussion

Our results suggest that M-TAPA may particularly provide somatic pain relief in six anesthetized dermatomes, including Th8–11, and long-term analgesic effect for up to 24 h postoperatively.

The incidence of venous thromboembolism (VTE) in patients with gynecological malignancies is reported to be 2.8–26.7% [13]. Prevention of VTE using a combination of sequential compression devices and low-molecular-weight heparin (LMWH) seemed suitable for gynecological cancer patients undergoing major abdominopelvic surgery; however, the removal of the epidural catheter may delay the initiation of LMWH therapy [14]. Epidural analgesia has been the gold standard in the treatment of perioperative pain after open gynecologic surgery; however, the associated side effects and complications, such as pruritus, prolonged block, and urinary retention, are a current concern. Thus, the importance of epidural anesthesia in gynecological surgery seems to be gradually diminishing [2, 15]. In our institution, before 2020, we used to perform multimodal analgesia including lateral TAPB without epidural anesthesia in gynecological surgery due to the initiation of anticoagulant therapy during the early hours of the POD 1. Radical hysterectomy often involves para-aortic lymphadenectomy and omentectomy, with an incision on the cranial side of the umbilicus. It is often necessary to consider the addition of subcostal TAPB or RSB, which requires multiple punctures because the analgesic range of the lateral TAPB cannot cover the incision above the umbilicus (Th10) [16]. Administration of a local anesthetic agent to multiple sites causes a decrease in the dose of the anesthetic agent in each site, which results in a short effective duration. The effectiveness of CWI through a multi-orifice catheter has also been reported [5, 6]. These studies reported the improvement of early postoperative NRS scores; hence, multi-orifice catheters may not provide even distribution from each pore. In other words, a multi-orifice catheter may function as a single-orifice catheter, except for the involvement of manual administration (≥ 270 mL/h) [7]. Quadratus lumborum block (QLB) was expected to provide “complete TAPB” due to the possibility of reducing visceral and somatic pain simultaneously in Th7–L2, and its effectiveness has been reported in gynecological surgery in recent years [17, 18]. However, it was indicated that the range of effects of QLB varies with individuals and that the expected effect may not be obtained with midline incision [19–21].

.M-TAPA was described by Tulgar et al. Several studies have reported the efficacy and duration of M-TAPA; however, the appropriate local anesthetic dose, effective duration, indications, and mechanism of action have not been investigated. Tulgar et al. initially reported an analgesic range of Th7–11 for M-TAPA with the administration of 25 mL 0.25% bupivacaine per side in laparoscopic ovariectomy and ileostomy [9]. Altiparmak et al. reported a Th5–10 range with 20 mL 0.25% bupivacaine in laparoscopic ventral hernia repair, while Aikawa et al. reported a Th3–12 range with 30 mL 0.25% ropivacaine in laparoscopic sleeve gastrectomy [10, 11]. We decided to use a local anesthetic amount of 30 mL (0.25% ropivacaine) per side if the patient weighed ≥50 kg and 25 mL (0.25% ropivacaine) if the patient weighed between 42 and 50 kg to certainly cover Th11–12. In our study, we obtained a 90% chance of analgesia in the Th11 area; hence, analgesia may not be certainly achieved in the Th12 area (60%). The present study result is in agreement with that of the pinprick test study by Aikawa et al. on laparoscopic gynecological surgery in terms of the certainty of the effect on Th8–10 in the anterior cutaneous branch region and the uncertainty of the effect on the lateral cutaneous branch region [12]. Similarly, the effect on the anterior cutaneous branch region Th12 could not be guaranteed, but we believe that the present study was more certain about Th11, as the previous study. This may be partly because they used 25 mL of local anesthetic in their study, whereas the present study used 30 mL of local anesthetic.

In addition, laparoscopic gynecological surgery is often performed in a head-down position, which may have affected the diffusion of the drug solution.

There remains a clinical question regarding the analgesic range of M-TAPA besides the certain effect in the Th11–12 area; it seems unclear why M-TAPA can block areas higher than Th9 without its injection medial to the linea semilunaris. In the subcostal TAPB, if Th6–8 coverage is desired, the local anesthetic should be injected medial to the linea semilunaris between the rectus abdominus muscle and the TAM because the linea semilunaris inhibits the spread of local anesthetic agents [16, 22]. We hypothesized that the existence of a ‘tunnel’ structure (the space between the costal cartilage and origin of the TAM) diffuses local anesthetic agents to the cranial side without the inhibition of the linea semilunaris. In the present study, we evaluated two cadavers. We investigated the spread of the dye at Th8–11. We changed the amount of injected dye from 25 mL to 30 mL for cadaver 2 because the dye did not spread to Th12 in cadaver 1. However, the increase in the volume of the dye seemed ineffective. We also confirmed the spread of the dye in our hypothesized “tunnel structure”, but we could not confirm the spread above Th7. This cadaveric study was performed only 30 min after the block was initiated in the supine position. The wide analgesic range reported in previous studies and in our study is likely to involve various factors, such as pneumoperitoneum, retractor, and intraoperative position, in addition to intraabdominal pressure in living humans. To prove the spread of the dye to the cranial side above Th7, further study under conditions closer to clinical conditions seems necessary.

Opioid consumption was commonly considered the primary outcome in recent studies. Nine patients discontinued IV-PCA within 48 h postoperatively, and this study did not significantly deviate from the total consumption expected from the baseline flow of IV-PCA. Considering the unstable effect in Th12 and the influence of visceral pain, the NRS score at 2 h postoperatively and opioid consumption are acceptable, and the patient satisfaction is relatively high. From the results of our study, it can be speculated that not only laparoscopic surgery but also surgery, requiring incisions above and below the umbilicus, such as open abdominal aortic replacement, may be a good indication. Contrarily, in lower abdominal surgery in which the wound is localized under the umbilicus, the indication must be carefully considered. In the lateral cutaneous branch innervation area, the pattern of analgesia was inconsistent, and further study is required to determine the indication for flank surgery.

There were some limitations to our study. First, the surgical procedure was not unified between the cases, and there are two patterns of skin incisions, from the pubis to above the umbilicus and from the pubis to the xiphoid process. Second, this was a single-center study with a small population because it was planned as a pilot study of a prospective study. Third, since IV-PCA was also used in this study and its effect on the pinprick test cannot be ignored, caution should be exercised in its interpretation. Finally, persistent postoperative pain and distant complications, such as ileus, were not assessed, and the quality of postoperative recovery was not evaluated.

## Conclusions

In conclusion, M-TAPA may provide an analgesic effect in approximately six dermatomes, including areas innervated by the anterior cutaneous branches of Th8–11, and long-term analgesia for up to 24 h postoperatively. Further studies on the mechanism of M-TAPA and comparison with other nerve blocks are required.

## Data Availability

The datasets used and/or analyzed during the current study are available from ‘The Dataset of Corresponding Author’.

## Abbreviations

VTE: Venous thromboembolism
TAPB: Transverse abdominis plane block
RSB: Rectus sheath block
QLB: Quadratus lumborum block
CWI: Continuous wound infiltration
TAPA: Thoracoabdominal nerves block through perichondrial approach
TAM: Transverse abdominis muscle
IV-PCA: Intravenous patient-controlled analgesia
POD: Postoperative day
NRS: Numerical rating scale
PONV: Postoperative nausea and vomiting
IQR: Interquartile range
LMWH: Low-molecular-weight heparin
